# Determine both the conformation and orientation of a specific residue in α-synuclein(61–95) even in monolayer by ^13^C isotopic label and p-polarized multiple-angle incidence resolution spectrometry (pMAIRS)

**DOI:** 10.1007/s44211-022-00128-0

**Published:** 2022-05-28

**Authors:** Chengshan Wang, Yiqun Zhou, Christopher Ewuola, Toyin Akinleye, Takeshi Hasegawa, Roger M. Leblanc

**Affiliations:** 1 Department of Chemistry, Middle Tennessee State University, 1301 East Main Street, Murfreesboro, TN 37132 USA; 2 Department of Chemistry, University of Miami, 1301 Memorial Drive, Coral Gables, FL 33146 USA; 3 Laboratory of Chemistry for Functionalized Surfaces, Division of Environmental Chemistry, Institute for Chemistry Research, Kyoto University, Gokasho, Uji, Kyoto 611-0011 Japan

**Keywords:** α-Synuclein, ^13^C amide I band, FT-IR, pMAIRS, Tilt angle, Conformation change

## Abstract

Protein’s magic function stems from its structure and various analytical techniques have been developed for it. Among proteins, membrane proteins are encoded 20–30% of genomes, whereas cause challenges for many analytical techniques. For example, lots of membrane proteins cannot form single crystal structure required by X-ray crystallography. As for NMR, the measurements were hindered by the low tumbling rates of membrane (i.e., phospholipid bilayers) where membrane proteins exist. In addition, membrane proteins usually lay parallel to the surface of phospholipid bilayers or form transmembrane structure. No matter parallel or perpendicular to phospholipid bilayers surface, membrane proteins form monolayer structure which is also difficult for X-ray and NMR to provide high-resolution results. Because NMR and X-ray crystallography are the two major analytical techniques to address protein’s structure, membrane proteins only contribute 2.4% to the solved protein databank. Surface FT-IR techniques can evaluate the conformation and orientation of membrane proteins by amide I band. Specifically for α-helical peptides/proteins, the orientation of the axis is critical to decide whether proteins form transmembrane structure. Notice that the traditional FT-IR can only provide “low-resolution” results**.** Here, ^13^C isotope was introduced into the nonamyloid component (NAC), which spans residues 61–95 of α-synuclein (α-syn). Then, p-polarized multiple-angle incidence resolution spectrometry (pMAIRS) was used to determine the orientation of a specific residue of α-helical NAC in monolayer. In general, pMAIRS is a novel technique to work complementary with X-ray and NMR to address membrane peptides/proteins structure with high resolution even in monolayer.

**Graphical abstract:**

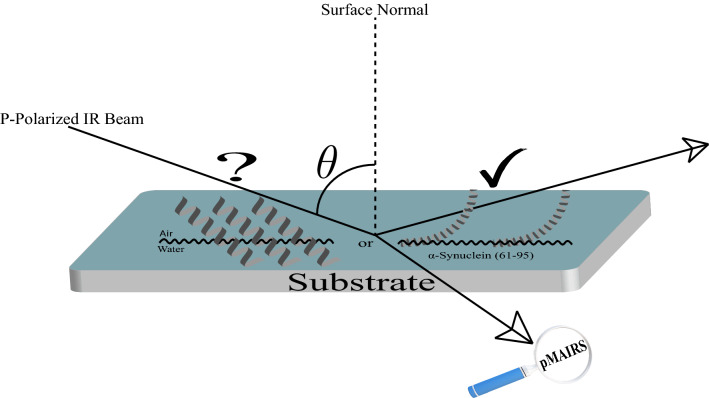

**Supplementary Information:**

The online version contains supplementary material available at 10.1007/s44211-022-00128-0.

## Introduction

**Scheme 1:**
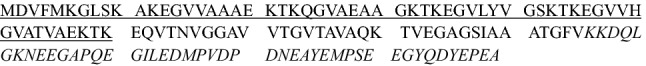
The sequence of α-synuclein with the N-terminus underlined and the C-terminus expressed in Italics

Among various motivations to develop analytical techniques to address protein’s structure [1–4], an important reason is that the malfunction of proteins causes many diseases. For example, Parkinson’s disease (PD) is hallmarked by the abnormal aggregation of α-synuclein (α-syn) which is a 140-amino-acid protein with its sequence shown in Scheme 1 [5] The primary structure of α-syn (Scheme 1) constitutes three domains: ^6^N-terminus (residues 1–60), the nonamyloid component (NAC) spanning residues 61–95, and C-terminus with residues 96–140 [6, 7]. Among the three domains, only the nonamyloid component (NAC) part (referred as α-syn(61–95) hereafter) is responsible for its aggregation [6]. In addition, considerable segmental peptides of α-syn have been detected in the lesion region in the PD patients brain [8]. Among the segment peptides, the NAC segment or α-syn(61–95) is an important one [8]. Especially, α-syn(61–95) has been detected to coaggregate with β-amyloid protein in the senile plaques in the brain of Alzheimer’s disease (AD) patients [9].

MDVFMKGLSK AKEGVVAAAE KTKQGVAEAA GKTKEGVLYV GSKTKEGVVH GVATVAEKTK EQVTNVGGAV VTGVTAVAQK TVEGAGSIAA ATGFV*KKDQL GKNEEGAPQE GILEDMPVDP DNEAYEMPSE EGYQDYEPEA.*

Although directly related to both PD and AD, the involvement of α-syn(61–95) in these diseases has been overlooked for a long time. Especially, the high-resolution result about α-syn(61–95) structure was limited, like many other membrane proteins [10, 11]. Among proteins, membrane proteins are reported to be encoded by ~ 20–30% of total genomes [10, 11]. Although various analytical techniques have been developed to address protein’s structure, membrane proteins cause challenges for the measurements via X-ray crystallography and NMR, which are the two major analytical methodologies able to provide high-resolution results about protein’s structure [1–4]. For example, many membrane proteins (including α-syn and α-syn(61–95)) cannot form single crystal structure required by X-ray crystallography [10, 11]. As for NMR, membrane proteins usually reside around cell membrane composed of amphiphilic phospholipid bilayer structure, which decreases the tumbling rates of NMR [11]. In addition, membrane proteins either form transmembrane structures or stay parallel to the surface of the amphiphilic phospholipids bilayer. Regardless of which above-mentioned structure is formed, the natural membrane proteins usually form a monolayer structure around cell membrane/vesicles. Since neither X-ray crystallography nor NMR can provides high-resolution results for proteins in the molecular monolayer structure, it is not a surprise that membrane proteins are reported to only contribute 2.4% to the solved protein databank [10, 11].

Thus, surface analytical techniques were developed to resolve this problem. Atomic force microscopy and scanning tunneling microscopy have been shown to be able to study the morphology of a monolayer [12, 13]. In addition, the thickness of a monolayer can be evaluated by ellipsometry and small-angle X-ray diffraction [14, 15]. To obtain the information about more detailed structure (such as secondary structure or conformation) of proteins/peptides in amphiphilic monolayer, surface FT-IR spectroscopy was used to address this issue by the characteristic peak position of amide I band [14], which stems from the stretching of the backbone carbonyls (i.e., C=O) in peptides/proteins. For example, the amide I band of β sheet is at ~ 1630 cm^−1^, while that of α-helix is between 1650 and 1660 cm^−1^ [16, 17]. In addition to secondary structures (i.e., conformations), surface FT-IR techniques was also used to determine the orientation (expressed by the tilt angle of a vibrational transition moment) [11, 14, 18], which is the key information to determine whether a membrane protein/peptide is transmembrane or not as mentioned above.

In general, surface FT-IR techniques include attenuated total reflection (ATR) [11], infrared external reflection spectroscopy (IR-ERS) [18], and p-polarized multiple-angle incidence resolution spectrometry (pMAIRS) [14]. Among these techniques, the available substrates for ATR are limited, while the signal of transition moments with tilt angle around the magic angle (i.e., 53.7°) cannot be detected by IR-ERS [18]. Therefore, IR-ERS can only qualitatively show whether a peptide/protein is roughly parallel or perpendicular to the interface [18]. As the most recently developed technique, pMAIRS can be used to accurately detect the orientation of various vibrations in ultrathin films (even monolayer structure) on a variety of substrates including CaF_2_ with a low refractive index [14]. By decomposing the spectrum to in-plane (IP) spectrum containing vibrations parallel to the interface and out-of-plane (OP) spectrum with perpendicular vibrations, the tilt angle of a vibration can be quantitatively determined by Eq. (1) shown in Materials and methods section in the Supplemental Information. To exhibit the above-mentioned character, IR-ERS and pMAIRS were compared by examining the orientation of α-syn and α-syn(61–95) because of their extensive interaction with membranes as discussed below [18, 19].

Although abundant in human brain, α-syn accumulates in the presynaptic terminals where high concentrations of vesicles exist [5, 6]. Thus, it is important for both AD and PD to understand the reason of the accumulation of α-syn and α-syn(61–95) in the presynaptic terminals and elucidate the structure as a monolayer at the amphiphilic interface. However, the reason of the accumulation of α-syn in the presynaptic terminals has been unclear due to the complication of the membrane structure. Fortunately, the amphiphilic membrane structure has been mimicked by the air–water interface as a simple model [18, 20], because the accumulation and the interaction between the proteins/peptides molecules at the interface can be precisely monitored by a Langmuir monolayer technique. Furthermore, the above-mentioned surface FT-IR techniques can be combined with Langmuir monolayer technique to address both the conformation and orientation of peptides/proteins at the interface [14, 18]. Thus, the conformation and the orientation of α-syn and α-syn(61–95) in the monolayer at the interface have been examined and compared as the following [18, 19].

α-Syn and α-syn(61–95) showed similar behavior at the air–water interface as the following [18, 19]. First, both α-syn and α-syn(61–95) formed a stable Langmuir monolayer at the air–water interface. Second, both of them were unstructured in aqueous solution and transformed to α-helical conformation at the interface [18, 19]. This conformation change and the consequent high stability of the monolayer at the interface may be responsible for the accumulation of α-syn and α-syn(61–95) at the interface [18, 19]. Finally, IR-ERS and pMAIRS were compared during the examination of the orientation of the axis of α-helical α-syn. IR-ERS could only qualitatively show that the axis of α-helical α-syn is roughly parallel to the interface [18]. In contrast, pMAIRS was shown to be able to quantitatively determine the tilt angle of the axis of α-helical α-syn(61–95) to be 30.1° [19]. The difference between IR-ERS and pMAIRS is due to the advantages (such as easy calculated tilt angle and capability to detect vibrations with tilt angle around 53.7°) of pMAIRS as mentioned above [18, 19].

**Scheme 2:**
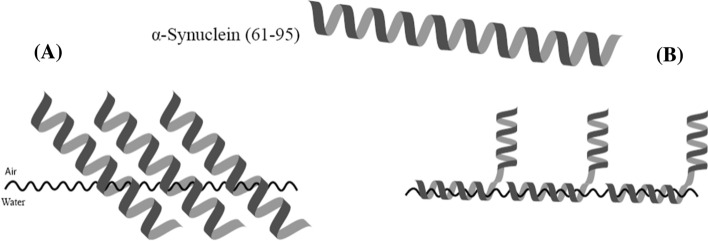
Illustration of two probabilities of α-syn(61–95) at the air–water interface

It is worth noting that the traditional FT-IR technique can only provide “low-resolution” results, which cannot be used to address the conformation or the orientation of a specific residue [21]. For example, a 0° tilt angle of a membrane protein means that the protein lies parallel to the membrane, whereas a 90° tilt angle shows that the protein forms a transmembrane structure. Therefore, 30.1° which is the average tilt angle of the axis at all the 35 residues in α-syn(61–95) causes confusions about the behavior of α-syn(61–95) at the interface: will it be parallel to the interface or form transmembrane structure? There are two likely answers to this question as shown in Scheme 2. One possibility is that the axis of all the 35 amino acid residues is 30.1° as shown in Scheme 2A. The other is that the axis of some residues is parallel, whereas that of other residues are perpendicular, as illustrated in Scheme 2B. The overall tilt angle of Scheme 2B is also around 30.1°. To address this issue, technique with higher resolution (such as residue-level resolution) result is needed.

Recently, the ^13^C isotope-edited FT-IR spectroscopy was developed to provide residue-level resolution by introducing ^13^C isotopic labels into the backbone carbonyl (i.e., C=O) of a peptide/protein [21, 22]. The ^13^C labeled C=O will generate a ^13^C amide I band which can provide the conformation of a specific residue [16, 17, 21]. It was reported that biophysical behavior (such as conformation and orientation) of residues close to the terminus might be different to the behavior of those in the middle of the sequence [16]. In this paper, a ^13^C isotopic label was introduced into the sequence of α-syn(61–95) in the backbone C=O of the glycine at position 93 (i.e., 93G), which is close to the C-terminus and serves as a proof-of-principle example here. pMAIRS was used to confirm that 93G is also in α-helix and determine the tilt angle of the axis at 93G of the α-helical α-syn(61–95) is almost at 0°, which means the parallel orientation of the axis at 93G to the interface. Therefore, the axis of some other residues than 93G must be more perpendicular to the interface, as shown in Scheme 2B. To the best of our knowledge, this is the first report to address both conformation and orientation of a specific residue of a membrane protein even in monolayer structure by pMAIRS and ^13^C isotope-edited FT-IR. The combination of these two techniques can serve as a novel analytical technique to supplement X-ray crystallography and NMR to address membrane protein structure with at least residue-level resolution even in monolayer. Detailed results are described below.

## Results and discussion

**Fig. 1:**
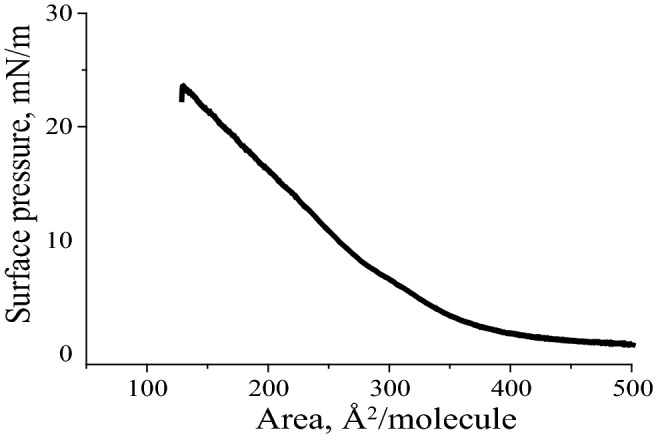
Surface pressure-area isotherm of the ^13^C labeled α-syn(61–95) at 93G

The details of the synthesis, purification, and Materials and Methods together with Mass result are described in the Supplemental Information, which (especially the Mass result in Fig. S1) confirms success of the synthesis and purification of the ^13^C labeled α-syn(61–95). First, the surface pressure-area (π–A) isotherm of the ^13^C labeled α-syn(61–95) at 93G is shown in Fig. 1. Similar to the previously published result of the unlabeled α-syn(61–95) [19], the lift-off point of the π–A isotherm was around 400 Å^2^/molecule. With the increment of surface pressure, the surface area decreases and the limiting molecular area was around 350 Å^2^/molecule. This similarity is reasonable, because the minor difference of replacing the ^12^C in the backbone carbonyl of 93G by ^13^C should not change the overall self-assembly behavior of α-syn(61–95), since it contains hundreds of carbons within it [19] (Fig. 1).

**Fig. 2:**
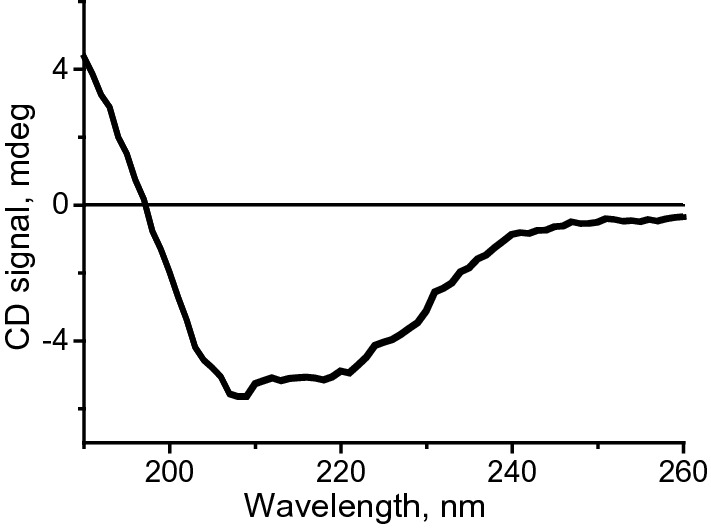
CD spectrum of the LB films of the ^13^C labeled α-syn(61–95) at position 93G on quartz slides transferred at 10 mN/m

**Fig. 3:**
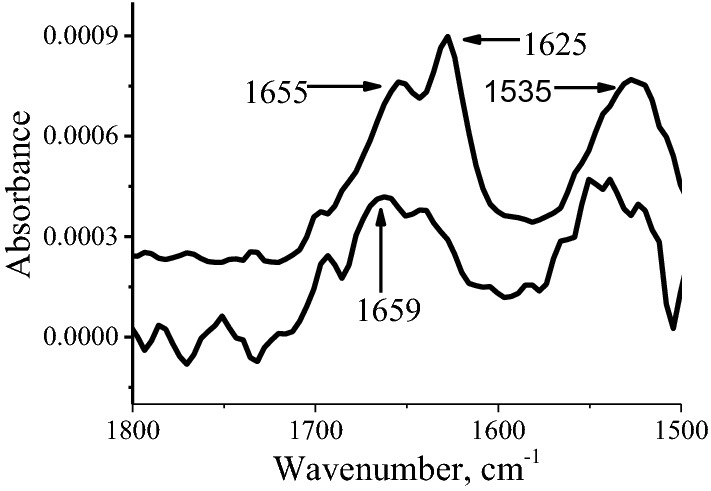
pMAIRS results of the LB monolayer of the ^13^C labeled α-syn(61–95) at position 93G prepared at 10 mN/m on silicon slide. The top curve is the IP spectrum and the bottom one is OP spectrum

In addition to π–A isotherm, circular dichroism (CD) was used to verify that the ^13^C labeled α-syn(61–95) also transformed to α-helix at the interface. As for the CD result of the Langmuir–Blodgett (LB) films of ^13^C labeled α-syn(61–95) on quartz slides shown in Fig. 2, two negative peaks were detected at 208 and 222 nm, together with the positive signal around 190 nm. Because all the peaks are the characteristic peaks of α-helix [19], the major conformation of α-syn(61–95) with ^13^C label at the interface is also α-helix. On the whole, both the π–A isotherm and the CD spectrum of the ^13^C labeled α-syn(61–95) are similar to those of the unlabeled peptide [19], because both techniques detect the overall biophysical behavior of α-syn(61–95). However, the pMAIRS results of the LB monolayer of the ^13^C labeled α-syn(61–95) are substantially different to those of the unlabeled peptide, as shown in Fig. 3.

The in-plane (IP) spectrum of the ^13^C labeled α-syn(61–95) is shown as the top curve in Fig. 3. Similar to that of unlabeled α-syn(61–95) published before [19], both regular amide I and II bands of α-helix were detected at 1655 and 1535 cm^−1^, respectively. However, a very strong ^13^C amide I band was also detected at 1625 cm^−1^ in the IP spectrum in Fig. 3. The peak at 1625 cm^−1^ cannot be assigned to the β-sheets conformation, because no signal of β-sheets was detected in the CD result (Fig. 2) mentioned above. In addition, it has been reported that the position of the ^13^C amide I band may be ~ 30–40 cm^−1^ lower than that of the regular amide I band of α-helix in the same environment [21]. Therefore, the position at 1625 cm^−1^ shows that the 93G is also in α-helix at the interface. In addition, the ^13^C amide I band in the IP spectrum is more intensive than the regular amide I band at 1655 cm^−1^, which is the sum absorption of all the other 34 residues in the sequence of α-syn(61–95). As described in the selection rule in previous publication,^14^ the peak intensity in pMAIRS can be affected by several factors such as thickness of the sample and the orientation of the transition moment. In the monolayer, the thicknesses of all the residues is almost the same and the orientation is the major factor to affect the peak intensity. Therefore, such an intensive ^13^C amide I band suggests a very small tilt angle (i.e., parallel orientation) of the ^13^C amide I transition moment.

The out-of-plane (OP) spectrum which is the bottom curve in Fig. 3 correlates to the IP spectrum and confirms this conclusion. The regular amide I and II bands were also detected in the OP spectrum. The regular amide I band splits slightly to 1659 and 1645 cm^−1^, possibly stemming from the coupling between the regular and the ^13^C amide I transition moment [11]. More importantly, the ^13^C amide I band at 1625 cm^−1^ was very weak in the OP spectrum (i.e., the *A*_OP_ is almost zero for Eq. (1) shown in Supplemental Information), even though the ^13^C label does exist at position 93G. According to the selection rule of pMAIRS shown in Eq. (1) in Supplemental Information, the tilt angle of the ^13^C amide I transition moment at 93G is ~ 0°, because the *A*_OP_ is almost zero.

As mentioned in the Introduction, the critical information for α-helical α-syn(61–95) at the interface is the orientation of its axis as shown in Scheme 2. Therefore, it is important to calculate the tilt angle of the axis by that of the amide I transition moment. The ^13^C amide I transition moment mainly stems from the stretching mode of ^13^C labeled backbone C=O, which was reported to be roughly parallel to the axis of the residue [19]. Therefore, the tilt angle of the axis of the α-helix at residue 93G may be equal to that of the ^13^C amide I transition moment, namely, ~ 0°, which means a defined parallel orientation of the axis at residue 93G. Since the overall tilt angle of the axis of α-helical α-syn(61–95) is 30.1°, some residues other than 93G may be more perpendicular to the interface as shown in Scheme 2B. On the other hand, pMAIRS may potentially provide structural results with even higher-level resolution in monolayer. This paper employed pMAIRS to reveal the tilt angle of the axis of α-helical α-syn(61–95) at 93G is ~ 0° by the ^13^C amide I band. Thus, the probability to screen the orientation of other specific transition moment by isotope labeling (such as replacing C–H in α-carbon in a specific amino acid by C–D where D is deuterium) in monolayer by pMAIRS cannot be ruled out. Therefore, higher than residue-level resolution (e.g., transition moment or chemical bond resolution) may be also obtained for membrane proteins even in monolayer by pMAIRS. As discussed in Introduction, the reason why X-ray crystallography and NMR are widely used for protein’s structure is due to their high resolution, which can reach atomic level for bulky samples [1–3]. Although representative surface techniques (e.g., atomic force microscopy, scanning tunneling microscopy, ellipsometry, and small-angle X-ray diffraction) can be used to study monolayer structure, the resolution of the results of these surface analytical techniques is usually limited for protein samples [12, 13]. As a consequence, pMAIRS works complementary to X-ray crystallography and NMR to address membrane protein structure with high resolution even in monolayer structure. In this aspect, pMAIRS will be soon employed as a bioanalytical tool especially for membrane proteins/peptides analysis.

Notice that the orientation of α-syn in various amphiphilic phospholipid bilayer structure has been studied, whereas controversial results have been published [23–27]. The reason of this controversy may stem from the complicated structure of the amphiphilic phospholipids bilayer with three detailed regions [20, 28], namely, the hydrophobic core, the hydrophilic headgroups, and the lipid-water interfacial layer. All the three regions can spontaneously affect the orientation of membrane proteins with lots of variable factors such as headgroup composition (e.g., positive or negative charged groups), alkyl chain structure of phospholipids (such as saturated and unsaturated alkyl chains), and so on. [20, 28] The air–water interface has been reported to be similar to the lipid-water interfacial layer, which generally exists around all the phospholipids bilayer with different characters to bulky water phase [29, 30]. Thus, the tilt angle of the axis of α-helical α-syn(61–95) at 93G may be ~ 0° in the lipid-water interfacial layer. It is worth noting that the results here do not contradict with any previous publications but serve as a control to evaluate the effect of the lipid-water interfacial layer on both the conformation and orientation of α-syn(61–95). In addition, the orientation may be affected by the presence of both headgroups and alkyl chains in phospholipid molecule, which can be also studied by pMAIRS in the future. Furthermore, pMAIRS can be also used to address the structure of α-syn(61–95) in the abnormal aggregates in β-sheet conformation, especially the early stage aggregation which embeds in the cell membrane and cause the death of the neuronal cells in the lesion part of PD patients as discussed in the Introduction [5–8]. As a consequence, the puzzle map of α-syn(61–95) in monolayer can be screened by pMAIRS with at least residue-level resolution to elucidate the pathology of both AD and PD.

## Conclusion

^13^C isotopic label was introduced into the backbone carbonyl of 93G in the sequence of α-syn(61–95). The ^13^C labeled α-syn(61–95) exhibits similar biophysical behavior to the unlabeled peptide at the air–water interface. For example, the ^13^C labeled α-syn(61–95) shows very similar π − A isotherm and CD spectrum to that of the unlabeled peptide and also forms α-helix at the interface. This similarity is reasonable, because only one ^13^C isotopic label should not change the overall biophysical behavior of α-syn(61–95), which contains hundreds of carbon atoms in it. On the other hand, the ^13^C isotopic label generated a new band (e.g., the ^13^C amide I band) in pMAIRS results, which quantitatively measured the tilt angle of the axis of α-helical α-syn(61–95) at 93G is ~ 0°. This indicates a very parallel orientation of the axis of the α-helix at 93G. Together with the overall tilt angle of the axis of the whole α-helical α-syn(61–95) around 30.1°, the axis at other positions than 93G (such as residues in the middle of the sequence) may be more perpendicular to the interface as shown in Scheme 2B. For the first time, the power of pMAIRS to accurately evaluate the tilt angle of the axis of α-helix in monolayer with residue resolution at the interface was exhibited. In general, pMAIRS can works complementary to X-ray crystallography and NMR to address the structure of membrane peptides/proteins with high resolution even in monolayer.

## Supplementary Information

Below is the link to the electronic supplementary material.

Supplementary file1 (DOC 190 KB)
