# Supplementary material for: Determine both the conformation and orientation of a specific residue in α-synuclein(61–95) even in monolayer by 13C isotopic label and p-polarized multiple-angle incidence resolution spectrometry (pMAIRS)
Source: Anal Sci. 2022 May 28;38(7):935–40. doi: 10.1007/s44211-022-00128-0 (PMC9206922; doi:10.1007/s44211-022-00128-0)
Supplement: Supplementary file 1 — Supplementary file1 (DOC 190 KB) [file 44211_2022_128_MOESM1_ESM.doc]

**Determine Both the Conformation and Orientation of a Specific Residue in α-Synuclein(61－95) Even in Monolayer by ^13^C Isotopic Label and p-Polarized Multiple Angle Incidence Resolution Spectrometry (pMAIRS)**

*Chengshan Wang, ** ^a^***^§^*** *Yiqun Zhou,*^b^***^§^*** *Christopher Ewuola,* ^a^ *Toyin Akinleye,* ^a^ *Takeshi Hasegawa,**^c^ *and Roger M. Leblanc**^b^

^a^ 1301 East Main Street, Department of Chemistry, Middle Tennessee State University, Murfreesboro, TN 37132, USA

^b^ 1301 Memorial Drive, Department of Chemistry, University of Miami, Coral Gables, FL 33146, USA

^c^ Laboratory of Chemistry for Functionalized Surfaces, Division of Environmental Chemistry, Institute for Chemistry Research, Kyoto University, Gokasho, Uji, Kyoto 611-0011, Japan

Supplemental Information

**Materials and Methods**

*Materials.* Diisopropylcarbodiimide (DIC), 1-hydroxylbenzotriazole (HOBT), Wang resin, and 9-Fluorenylmethoxycarbonyl (Fmoc) protected amino acids used for peptide synthesis were purchased from Thermo Fisher Scientific Inc. (Pittsburgh, PA). All of the amino acids used were in L-configuration, except for glycine. Trifluoroacetic acid, piperidine, and organic solvents (such as acetonitrile, N,N-dimethylformamide or DMF, dimethyl sulfoxide or DMSO, and etc.) were also from Fisher Scientific. The ^13^C isotopic labeled Fmoc-glycine was purchased from AnaSpec Inc. (Fremont, CA). Quartz slides, which were used for Langmuir-Blodgett (LB) film deposition of ^13^C labeled α-syn(61-95) with a dimension of 1.0 × 4.0 cm^2^ for circular dichroism measurements, were purchased from Hellma Cells Inc. (Plainview, NY). The silicon wafer for the LB monolayer deposition was from University Wafer Inc. (Boston, MA). All aqueous solutions were prepared using Millipore water (18 MΩ•cm).

*Peptide Synthesis and Purification.* Same as then unlabeled peptide,^1^ peptide of ^13^C labeled α-syn(61－95) was synthesized by solid phase (Fmoc) chemistry. DIC and HOBT were used for the coupling reactions of the Fmoc protected amino acids. The coupling of all the residues in the sequence utilized Fmoc protected unlabeled amino acids except 93G, which used a Fmoc-Gly with a ^13^C labeled carbonyl. After coupling, the Fmoc groups were removed by a 20% piperidine solution in DMF (v/v). After all the 35 residues were coupled and deprotected, the peptide was cleaved from the resin by trifluoroacetic acid. Then, the crude product was purified by semipreparative reversed-phase high-performance liquid chromatography (RP-HPLC) on a Waters Breeze 2 separation system equipped with a 1525 EF binary pump and a column (Jupiter-10-C18-300, 10 mm i.d. × 250 mm) from Phenomenex (Torrance, CA). The mobile phases included: 0.1% trifluoroacetic acid in water (v/v, mobile phase A) and 0.1% trifluoroacetic acid in acetonitrile (v/v, mobile phase B). The elution gradient used 10−45% B for 40 min at a flow rate of 4.7 mL/min. The success of the synthesis and purification were confirmed by fragmental MS from a Waters SYNAPT q-TOF tandem mass spectrometer as discussed below.

*Surface Chemistry Study.* The surface pressure-area (π−A) isotherm of the Langmuir monolayer of the ^13^C labeled α-syn(61－95) was conducted in a A Kibron µtrough (Kibron Inc., Helsinki, Finland), as well as the deposition of its LB film to quartz or silicon slides. The concentration of the ^13^C labeled α-syn(61－95) aqueous solution was 0.25 mg/mL. 25 µL of the ^13^C labeled α-syn(61－95) solution was spread at the air-water interface, followed by a 30-min period for the monolayer formation. The LB films of α-syn(61－95) were then made by transferring the ^13^C labeled α-syn(61－95) Langmuir monolayer to quartz slides and silicon wafer under the surface pressure 10 mN/m (as suggested in figure caption). After held for two hours before the transference, the quartz slide and silicon wafer was moved at 0.3 cm/minute.

*Circular Dichroism and Mass Measurement.* The circular dichroism (CD) spectra were measured by a JASCO J-810 spectropolarimeter. The CD spectrum was recorded with a response time of 8 s and a scan speed of 20 nm/minute. Six quartz slides with the LB film on them were stacked for the CD measurement. The success of the synthesis of ^13^C labeled α-syn(61－95) was confirmed by a Waters SYNAPT q-TOF tandem mass spectrometer, which was equipped with electron spray ionization and APCI ion source. The sample cone temperature was 150 °C and the capillary voltage was 3000 V. 500 L/h N_2_ was used to evaporate the ^13^C labeled α-syn(61－95) aqueous solutions, which contains 0.1 % of trifluoroacetic acid. Positive ion mode was used for MS and MS/MS measurements. As for fragment MS/MS measurement to confirm the sequence of the ^13^C labeled α-syn(61－95), the high purity of argon was used as collision gas and the collision energy of 4500 V was used.

*p-Polarized Multiple-Angle Incidence Resolution Spectroscopy (pMAIRS) Measurements***.** pMAIRS measurements were performed on the Nicolet iS50 FT-IR spectrometer (Thermo Fisher Scientific, Madison, MA) equipped with a pMAIRS accessory. The LB film monolayer on the silicon (Si) substrate was put in the pMAIRS accessory and the IR beam transmitted through the sample. The refractive IR beam was led to a HgCdTe (MCT-D*) detector cooled by liquid nitrogen. The bare Si substrate was used as the background for measuring the spectra of the ^13^C labeled α-syn(61－95) sample. The modulation was 50 kHz. The spectra of both background and sample were acquired over five thousand scans at a resolution of 8 cm^-1^, under the optimal conditions for a Si substrate.[^1^](#_ENREF_1) The in-plane (IP) and out-of-plane (OP) spectra were calculated based on all the background and sample results of various incident angles from 9º to 44º. The orientation angle (i.e., $\varphi$in equation 1 below) of the amide I band in relation to the surface normal was calculated the following equation:^2^

$\varphi=\tan^{-1}\sqrt{\frac{2A_{IP}}{A_{OP}}}$ Equation 1

Where A_IP_ represents the peak area of amide I band in the IP spectrum while the A_OP_ is that of the amide I band in OP spectrum. The tilt angle of the amide I transition moments in relation to the surface is equal to 90º minus $\varphi$ (i.e., 90º - $\varphi$). All the data in this manuscript has been repeated for at least three times.

**Mass result of ^13^C labeled α-syn(61－95).** The electrospray Mass spectra of the purified α-syn(61－95) with ^13^C labeled carbonyl of 93G is shown in Figure S1. The peak appearing at 1631.92 is assigned to the diprotonated α-syn(61－95) and the peak at 1088.28 is triple protonated α-syn(61－95). Based on the values above, the molecular weight of α-syn(61－95) with ^13^C label at the backbone carbonyl of 93G is 3261.8 Da which measured by Mass is very close to theoretical value at 3261.6 Da. Compared with the result of unlabeled α-syn(61－95), the peak at 1631.92 is more intensive and the quadruple protonated peak around 817 disappeared.^1^ This is because of the less acids mixed with the sample during the Mass measurement. In general, the synthesis and purification of α-syn(61－95) with ^13^C label is successful.

Figure S1. Mass spectrum of α-syn(61－95) with ^13^C label in the backbone carbonyl of 93G

Reference

1. Wang, C.; Sharma, S. K.; Olaluwoye, S. O.; Alrashdi, S. A.; Hasegawa, T.; Leblanc, R. M., Conformation change of α-synuclein(61－95) at the air-water interface and quantitative measurement of the tilt angle of the axis of its α-helix by multiple angle incidence resolution spectroscopy. *Colloid. Surf. B* **2019,** *183*, 110401.
2. T. Hasegawa*, Quantitative Infrared spectroscopy for understanding of a condensed matt*er, Springer 2017.
